# Computational analysis of morphological and molecular features in gastric cancer tissues

**DOI:** 10.1002/cam4.2885

**Published:** 2020-02-03

**Authors:** Yoko Yasuda, Kazuaki Tokunaga, Tomoaki Koga, Chiyomi Sakamoto, Ilya G. Goldberg, Noriko Saitoh, Mitsuyoshi Nakao

**Affiliations:** ^1^ Department of Medical Cell Biology Institute of Molecular Embryology and Genetics Kumamoto University Kumamoto Japan; ^2^ Department of Health Science Faculty of Medical Science Kyushu University Fukuoka Japan; ^3^ Image Informatics and Computational Biology Unit Laboratory of Genetics National Institute on Aging National Institutes of Health Baltimore MD USA; ^4^ The Cancer Institute of JFCR Tokyo Japan

**Keywords:** ATF7IP/MCAF1, gastric cancer, machine learning, PD‐L1, tissue morphology

## Abstract

Biological morphologies of cells and tissues represent their physiological and pathological conditions. The importance of quantitative assessment of morphological information has been highly recognized in clinical diagnosis and therapeutic strategies. In this study, we used a supervised machine learning algorithm *wndchrm* to classify hematoxylin and eosin (H&E)‐stained images of human gastric cancer tissues. This analysis distinguished between noncancer and cancer tissues with different histological grades. We then classified the H&E‐stained images by expression levels of cancer‐associated nuclear ATF7IP/MCAF1 and membranous PD‐L1 proteins using immunohistochemistry of serial sections. Interestingly, classes with low and high expressions of each protein exhibited significant morphological dissimilarity in H&E images. These results indicated that morphological features in cancer tissues are correlated with expression of specific cancer‐associated proteins, suggesting the usefulness of biomolecular‐based morphological classification.

## Graphical abstract

A machine learning algorithm wndchrm measures and classifies morphological features of gastric cancer tissues, which are correlated with the expression of specific cancer‐associated proteins.

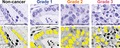

## 1 INTRODUCTION

It is essential to quantitate morphological and molecular features of cells and tissues under physiological and pathological conditions. In particular, various cellular and noncellular components, which may include currently unknown ones, coexist at the same and adjacent sites in tissues, resulting in spatiotemporal heterogeneity. Furthermore, each cell unit possesses the nucleus and cytoplasm, which structurally and functionally cooperate for gene expression and cellular dynamics.^1^, ^2^ These components systematically orchestrate and dynamically change in a variety of disease states such as cancer.^3^, ^4^ Characteristics of cancerous tissues derived from patients provides diagnostic information regarding the tumors, and allows prediction of therapeutic responses^5^, ^6^ and prognosis.^7^, ^8^, ^9^ Pathological assessment of cancer specimens stained with hematoxylin and eosin (H&E) is primarily interpreted not only by tissue architecture but also by nuclear morphology of the tumor cells, which has been used for routine clinical diagnosis^4^, ^10^ and computer‐aided pathological diagnosis.^11^, ^12^, ^13^ Recently, this field has significantly progressed to decipher clinical and biological relevance from such pathological images by combining molecular information such as genomic data.^14^, ^15^

Gastric cancer is one of the most common human cancers, and is the second leading cause of cancer‐related deaths worldwide.^16^, ^17^ As it is often associated with chronic inflammation caused by *Helicobacter pylori* infection and chemicals,^18^ this disease is an example of human oncogenesis that is etiologically induced by environmental factors.^19^, ^20^ Thus, gastric cancer is heterogeneous with distinct clinical phenotypes at diagnosis, differing responses to treatment, and subsequent prognosis. Despite preventive strategies and many therapeutic efforts, gastric cancer is often diagnosed at advanced stages. Histologically, the majority of gastric cancers are adenocarcinomas, which stem from the glands of the stomach, and are classified into two major types, “differentiated and undifferentiated types” and “Lauren intestinal and diffuse types”.^21^, ^22^ It is crucial to understand the histological and molecular basis of gastric cancer to identify diagnostic and therapeutic targets involving this disease.

Mathematical instructions, including machine learning or deep learning algorithms, can quantitatively classify morphological features or detect histological components such as cell nuclei, lymphocytes and stroma in complex tissue spaces.^23^, ^24^, ^25^ Although current studies have shown good correlations between morphological differences and patient prognoses, it is still challenging to further improve computational strategies. Among these, weighted neighbor distances using a compound hierarchy of algorithms representing morphology, shortly the *wndchrm* (weighted neighbor distances using a compound hierarchy of algorithms representing morphology),enables classification and mining of images to identify similarities or dissimilarities, without predefining target morphological features.^26^, ^27^ *Wndchrm* computes a large number of image features and extracts effective ones to discriminate between classes by calculating Fisher Discriminant scores, together with measuring classification accuracy and morphological dissimilarity. This approach has been previously applied for diverse set of images: characterization of muscular deficiencies in physiological aging in *C elegans*,^28^ detection of morphological differences of osteoporosis in human knee X‐ray images,^29^ and assessment of melanoma progression by tissue microarrays stained with H&E.^30^ Using *wndchrm*, we have classified normally or abnormally reprogrammed human‐induced pluripotent stem (iPS) cells by measuring morphological differences in colony formation and nuclear subdomains such as the promyelocytic leukemia (PML) nuclear bodies.^31^ We have also measured morphological changes of the nucleolus and mitotic chromosomes upon depletion of the cellular components in cell lines.^32^, ^33^

Here using *wndchrm*, we quantitatively investigated morphological features and classification of gastric cancer tissues that included heterogeneous cell populations. Our results indicated that *wndchrm* reliably computes morphological changes of tumors with differentiation grades, and that cancer‐associated protein‐based analysis emphasized a correlation between molecular expression and tissue structures.

## 2 MATERIALS AND METHODS

### 2.1 Histopathological specimens

Human gastric tissue microarray, and paraffin‐embedded gastric tumor and nontumor samples were purchased from BioChain Institute (catalogue number:Z7020045), ISU ABXIS Co., Ltd (catalog number: #112110611141), ZYMED Laboratories (catalog number: 75‐4013), ILSbio LLC (catalog number: ILS34202PD2) and US Biomax, Inc (catalog number: HStm‐Ade180Sur‐02). There were 66 stomach adenocarcinoma tissues with diagnostic results. We used histological grading with reference to a datasheet and the classification.^21^, ^22^ Donor information is summarized in Table S1, S4, and S5. The formalin‐fixed tumors were processed for paraffin‐embedding and sliced to 4‐µm thick sections with a microtome (Leica RM2125RT), and were subjected to H&E staining.

### 2.2 Immunohistochemistry

Immunohistochemistry (IHC) for ATF7IP/MCAF1 and PD‐L1 were performed with human gastric paraffin‐embedded tissues (ILSbio, LLC) and gastric tissue array (US Biomax, Inc). The array slides were deparaffinized using xylene and ethanol, and then incubated in methanol with 3.0% hydrogen peroxide for 30 minutes to block endogenous peroxidase activity. The tissue sections were boiled for 10 minutes at 120°C in an autoclave in citrate buffer (ethylenediaminetetraacetic acid for PD‐L1) for antigen retrieval. For PD‐L1 IHC, antigen retrieval was processed before blockade of endogenous peroxidase activity. The sections were immersed in 1.0%Block Ace (Dainippon Sumitomo Pharma Co., Ltd.) in phosphate‐buffered saline for 60 minutes, then incubated with anti‐ATF7IP/MCAF1 or PD‐L1 antibodies overnight at 4℃. The immunoreaction was visualized using Histofine Simple Stain MAX‐PO (Nichirei Bioscience) and 3,3‐diaminobenzidine tetrahydrochloride (DAB) (Agilent Dako). The slides were counterstained with hematoxylin and mounted with Malinol (Muto Pure Chemicals).

### 2.3 Image capture and processing

Original images from H&E or IHC slides were obtained under a microscope (BX51; Olympus) equipped with a UPlan SApo 20× objective lens through a digital camera (DP72; Olympus). All images were captured as 24‐bit color and 1360 × 1024 pixels. The color deconvolution plugin in Image J software (http://imagej.nih.gov/ij/) was used to separate H&E images into the H and E channels,^30^ or IHC images into DAB and hematoxylin channels. The mean intensity per image for DAB signals was calculated with Image J software. The images from the DAB channel were inverted, and then the mean intensity per image for DAB signals was calculated.

### 2.4 Quantitation of morphological differences

Morphological differences were measured with the*wndchrm* algorithm (*wndchrm* ver1.52).^26^, ^27^ Numbers of the ratio of test to total images were 33% in most analyses. Images were tiled as −t1 (no tiling), −t2 (into 4 images), −t4 (into 16 images), −t6 (into 36 images), −t8 (into 64 images) and, −t10 (into 100 images) and −t12 (into 144 images). Cross‐validation tests were automatically repeated 20 times to validate classification performance. As described previously,^34^, ^35^ dendrograms and morphological distances were identified using the Fitch‐Margoliash method and calculating Euclidian distances (d = √Σ(A−B)^2^), respectively. Fisher scores were computed from 4,059 (24‐bit color) or 2919 (8‐bit gray) image features from the following: Chebyshev‐Fourier transform (ChFT), Chebyshev Statistics (Ch), Combined First Four Moments (Cf4M), Fractal Statistics (Fra), Haralick Texture (Har), Multiscale Histogram (MSH), Radon (Rad), and Zernike (Zer), and the others (others).^26^, ^27^ For evaluation of nuclear morphology, hematoxylin‐stained images were analyzed with a Cellomics CellInsight with HCS studio cell analysis software (ThermoFisher Scientific) (Figure 3).

### 2.5 Antibodies

The rabbit anti‐ATF7IP/MCAF1 polyclonal antibody (used at 1:100)^36^ and rabbit anti‐PD‐L1 polyclonal antibody (at 1:75, E1J2J, Cell Signaling Technology) were used for IHC.

### 2.6 Data analysis

R software version 3.1.3 was used for statistical analysis, F‐test for the equality of two variances, Student's *t* test, and Welch's *t* test for the means of two populations were used for two variances. The Pearson correlation coefficient was utilized to evaluate the similarity of image features. For evaluation of classification performance, the pROC‐package was used for plotting receiver operating characteristics (ROC) curves and calculating area under the receiver operating characteristic curves (AUCs). Sensitivity was calculated as (true positive)/(true positive + false negative) for cancer classification or high expression of molecular markers, and specificity was calculated as (true negative)/(true negative + false positive) for instances of noncancer or low expression. We calculated 95% confidence intervals using binomial tests.

## 3 RESULTS

### 3.1 *Wndchrm*‐based analysis of morphology in noncancerous and gastric cancer tissues

**Figure 1:**
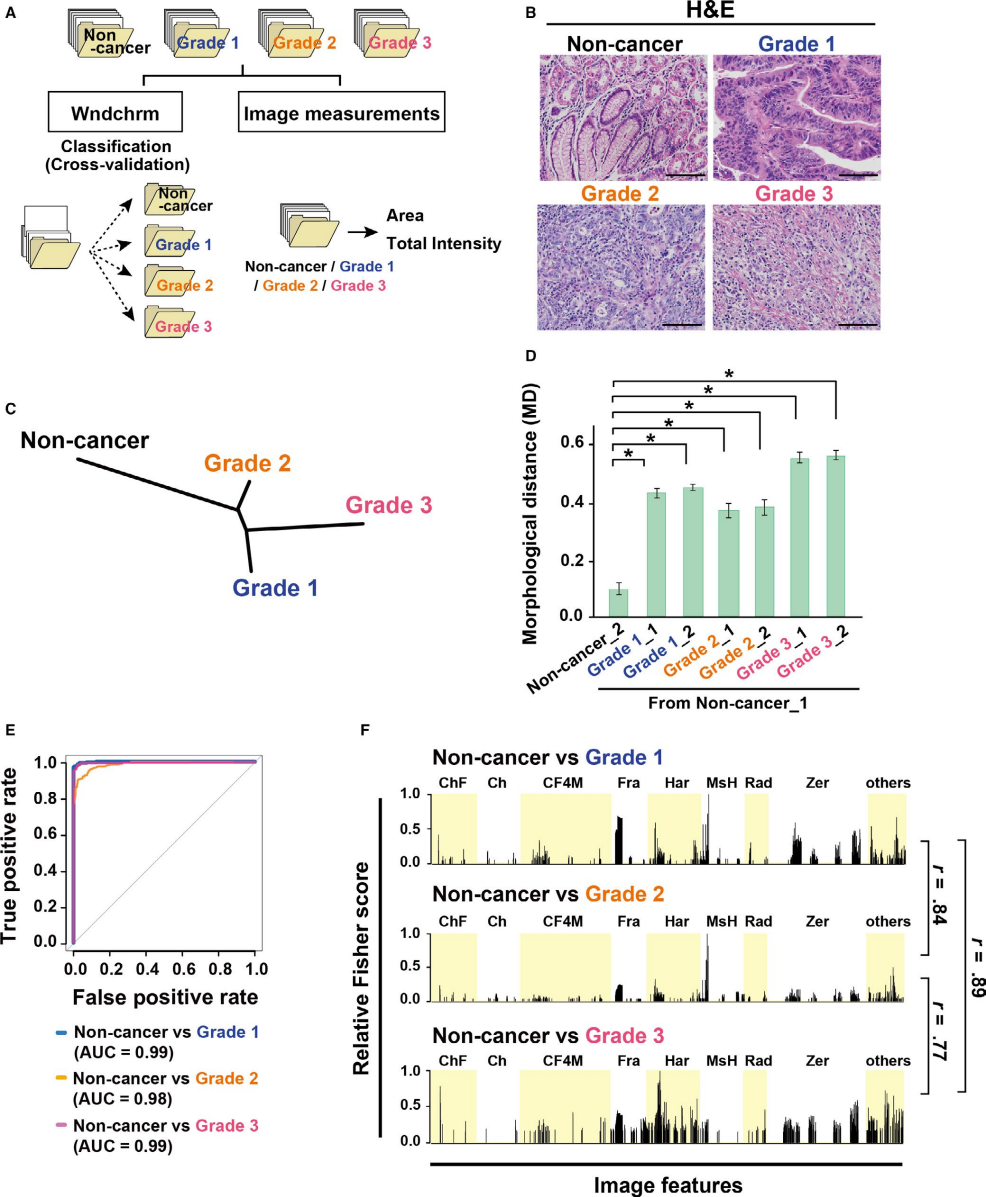
*Wndchrm* algorithm quantitatively classifies morphological differences between noncancer and gastric cancer tissues. A, The scheme for computational image analysis of morphology in gastric cancer tissues. Classification of tissue morphology by *wndchrm* (*left*), and image measurements (*right*). We used the histological grade in gastric cancer tissues tested, with reference to a datasheet and the classification:^21^, ^22^ Non‐cancer, Grade 1 (well differentiated), Grade 2 (moderately differentiated), and Grade 3 (poorly differentiated). B, Representative H&E images of noncancer and gastric cancer tissues used for classification in (C‐F). Scale bar, 100 µm. C, A dendrogram made with the cross‐validation tests shows morphological similarity (n = 54 images in each class). D, Morphological distance (MD) from noncancerous gastric tissue Non‐cancer_1. One class was randomly divided to two subclasses (for example, Non‐cancer_1 and Non‐cancer_2; n = 27 images in each subclass). *P* values were calculated using Student's *t* test or Welch's *t* test (*P < .001). C, ROC curves derived from the binary classification (n = 54 images in each class; classification accuracy shown in Figure S1D). F, Typical relative Fisher discriminant scores assigned to the 4059 features for binary classification in (E). Peaks represent image features that were useful for the indicated classifications. The maximum Fisher score was set to 1. Image features useful for classification were highly correlated among each test (r > 0.7)

To quantitatively assess biological morphology of cell and tissue conditions, we performed a machine learning analysis using the *wndchrm* algorithm, and specific image measurements (Figure 1A). Image data‐sets were constructed in accordance with pathological diagnosis, using tissue microarrays derived from human stomach adenocarcinoma patients. Fifty‐four H&E‐stained tissue images of 1360 × 1024 pixels were collected for each class: Noncancer, Grade 1 (well differentiated), Grade 2 (moderately differentiated), and Grade 3 (poorly differentiated) (Figure 1B, Figure S1A and Table S1). Briefly, *wndchrm* extracted image features from all images of each defined class, and trained a classifier to discriminate between the classes using training data‐sets. The classification performance was then validated with test images that were randomly selected, where these steps were automatically performed. We carried out 20 cross‐validation analyses among the noncancer and grades 1‐3 of gastric cancer (Figure 1A, *left*). As an initial step of the analysis, we examined the optimal number of images necessary for efficient classification. The results showed that the value of classification accuracy (CA) improved with increasing numbers of training images (Figure S1B), while that of standard errors became smaller as often seen in machine‐learning analyses.^31^The best classification was found with 54 training images at CA 0.78 (the maximum CA is possibly 1.0), and this CA value was markedly higher than random classification at CA 0.25. Furthermore, the relative similarities among the classes were visualized with dendrograms (Figure 1C). In addition, using 20 images in each classes, we confirmed the classification similarity between noncancer, chronic gastritis and grades 1‐3 (Figure S1C). Performance of the classification test was sufficient, as its specificity and sensitivity to discriminate cancer grades from noncancerous tissues was 100% and 92%, respectively (Table S2, *upper*). For additional assessment of morphological features, we divided each class into two subclasses, and measured the degree of dissimilarities of grades 1‐3 from noncancerous tissues, as indicated by morphological distance (MD) (Figure 1D). The MD from Non‐cancer_1 showed similarity to Noncancer l_2 and dissimilarity to cancer tissues of three grades. Furthermore, when the images were digitally tiled (see Methods), the numbers of training images increased, but the overview of the tissues was lost. However, CA values were largely unchanged at 0.79‐0.69 using these tiled images (Figure S1D), suggesting that local morphology as well as histological overview are indicators to discriminate between noncancer and cancer tissues.

We then performed detailed binary comparisons between noncancer samples and each grade of gastric cancer to evaluate the effectiveness of *wndchrm* using each of the 54 images that showed sufficient CA values (Figure S1E). ROC curve analyses verified the accuracy of classifications, because AUCs were 0.99, 0.98, and 0.99 for Noncancer versus Grades 1, 2, and 3, respectively (the maximum AUC is 1.0, in contrast to random assignment of 0.5) (Figure 1E). Representative lists of informative image features in each classification test were indicated according to relative Fisher discrimination scores (Figure 1F). Many sets of image features were commonly used to discriminate Grades 1, 2, and 3 from Non‐cancer (r > 0.7), although some distinct features were also involved (data not shown). Our results showed that *wndchrm* analyses highly recapitulated the human‐based pathological examinations of H&E images of cancer tissues.

### 3.2 *Wndchrm*‐based analysis reveals informative features of H&E‐stained images

**Figure 2:**
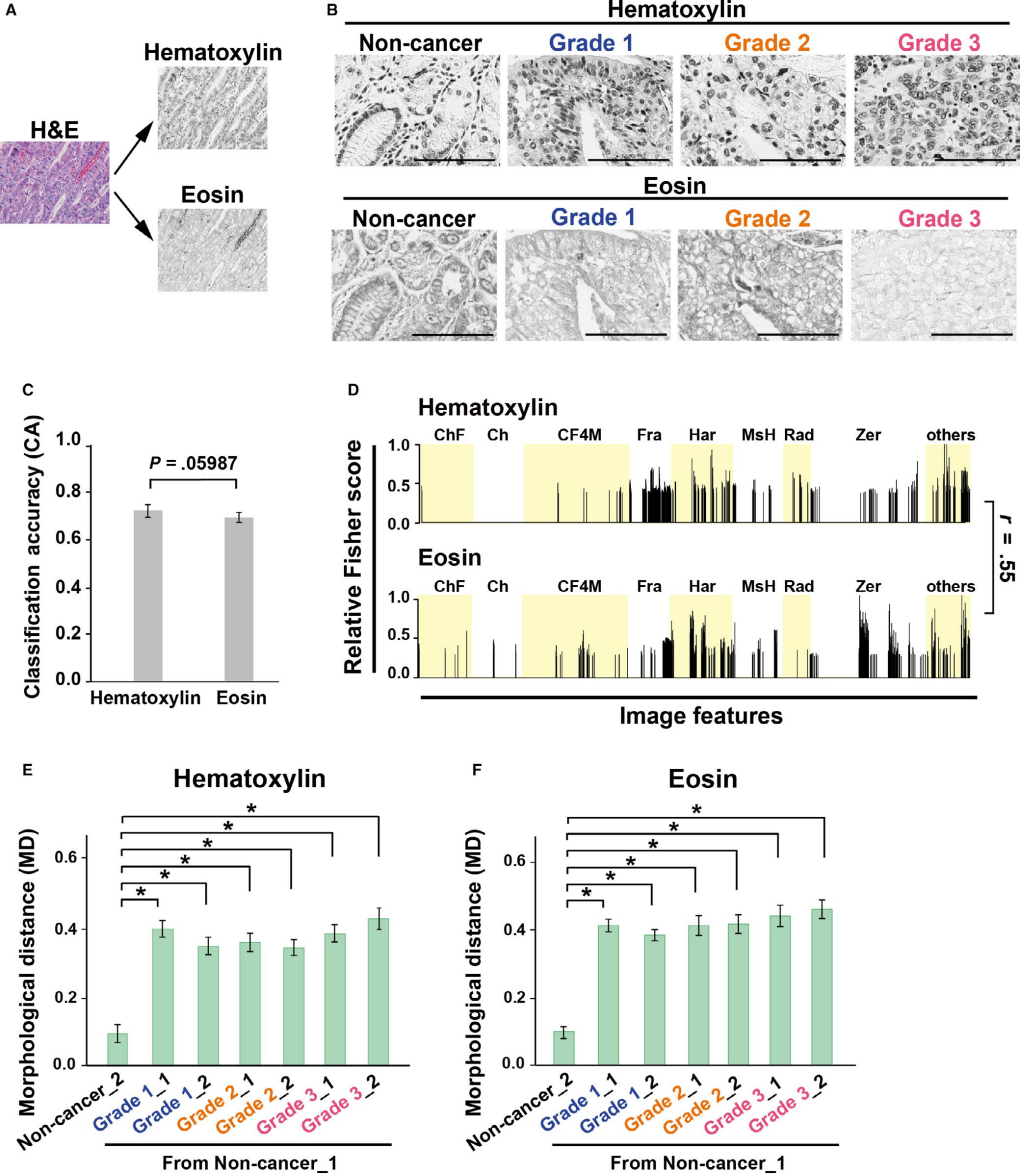
Nuclear and cytoplasmic images are informative for morphological classification of gastric cancer tissues. A, H&E images were digitally split into hematoxylin‐ and eosin‐stained image channels. B, Representative images used for wndchrm analysis in (C‐F). Scale bar, 100 µm. C, The CA among noncancer and Grades 1‐3 which were measured with hematoxylin‐ and eosin‐stained images (n = 54 for each class). *P* values were calculated using Student's *t* test. D, Relative Fisher discriminant scores assigned to the 2919 features for each test in **c**. The Pearson correlation is considered to be weak (r = 0.55). E and F, MDs of each subclass from noncancerous gastric tissue (Non‐cancer_1), using hematoxylin (E) and eosin‐stained images (F). *P* values were calculated using Student's *t* test or Welch's *t* test (*P < .001)

To understand which morphological features contribute to classification of noncancer and cancer grades, we digitally deconvolved the H&E RGB (Red, Green, Blue) images into hematoxylin and eosin channels in gray scales (Figure 2A).^30^ Cellular nuclei and cytoplasmic components are generally stained with hematoxylin and eosin, respectively.^4^, ^10^ Using the deconvolved images for noncancer and grades 1‐3, as shown in Figure 2B and Figure S2, we measured CA among noncancer and grades 1‐3 (Figure 2C). Cross‐validation tests of hematoxylin and eosin images indicated equivalent CA values (0.72 and 0.69, respectively). Sensitivity and specificity were equally high at 82%‐98% (Table S2, *second from upper*), suggesting that hematoxylin and eosin images contain morphological features distinguishing between cancer and noncancerous tissues.

A typical list of informative image features in the classification test was created according to relative Fisher discrimination scores, and showed overall similarities (Figure 2D). Pearson correlation coefficient value was weak between hematoxylin and eosin images (r = 0.55), suggesting the presence of unique morphological characteristics in either image. Consistently, MDs from Non‐cancer_1 in both hematoxylin and eosin images showed dissimilarity between noncancerous and cancer tissues to a similar extent (Figure 2E,F).Thus, *wndchrm* analyses implied the presence of informative features in hematoxylin and eosin‐stained images of cancer tissues.

### 3.3 Characterization of nuclear morphology in gastric cancer tissues

**Figure 3:**
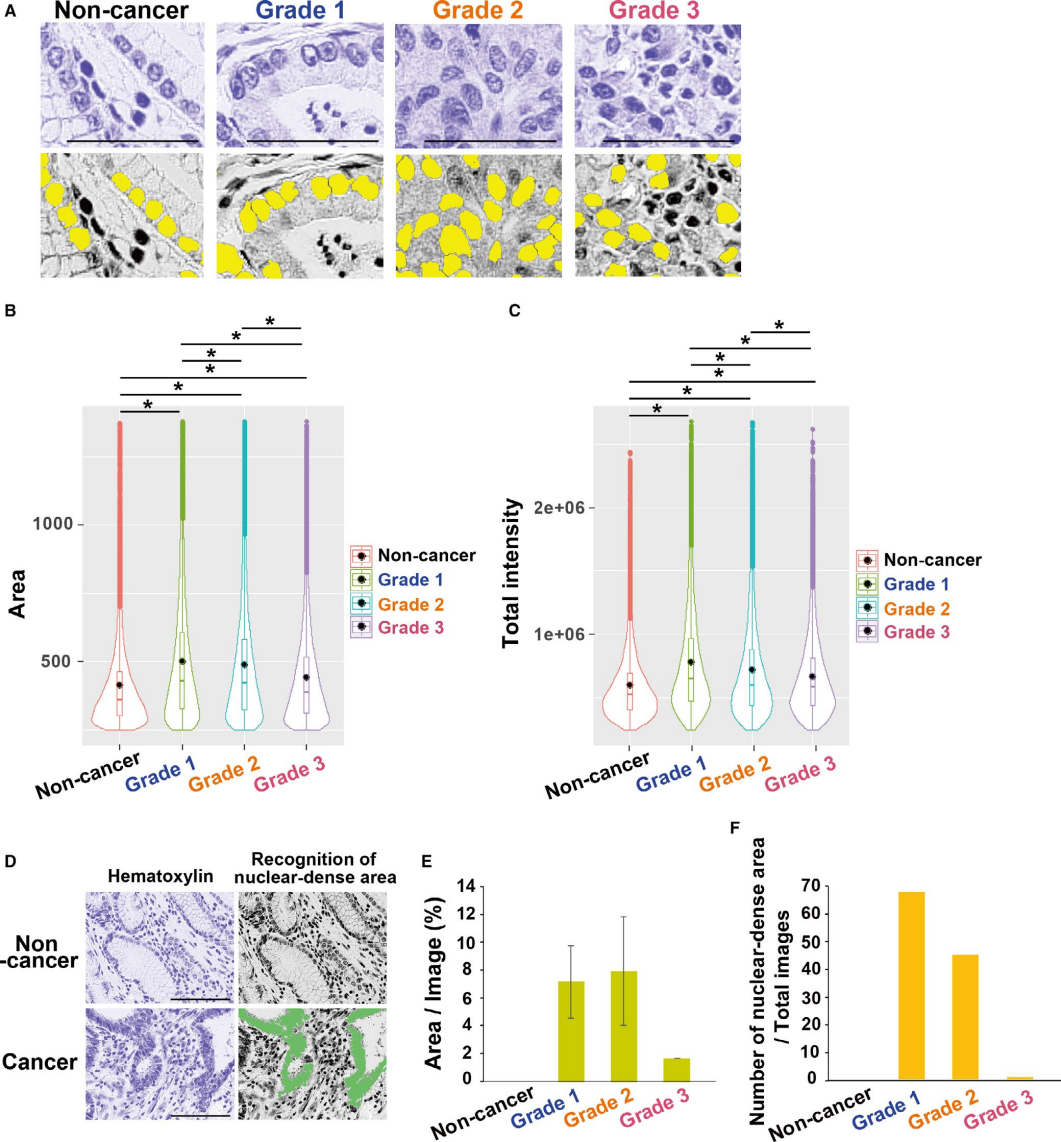
Analysis of nuclear morphology and densities in gastric cancer tissues. A, Original hematoxylin‐stained images (top), and automatic detection of nuclei indicated with filled yellow markers (bottom). The nuclei were recognized with CellInsight software. Scale bar, 50 µm. B and C, Violin plot for the nuclear area (B) and total staining intensities (C). The counted nuclei were as follows; 12,193, 14,811, 15,157, and 16,379 for Non‐cancer, Grade 1, Grade 2, and Grade 3, respectively. In the violin plot, the box bar in the center represents the interquartile, and the inside line and black dot show median and average values, respectively. *P* values were calculated using Welch's *t* test (*P < .001). D, Representative images of automatically segmented nuclear‐dense areas. Massive nuclear clusters were detected as continuously hematoxylin‐positive areas which were larger than the defined threshold (>13 200 pixels and filled‐green). E, The average occupancies (%) of the nuclear dense areas per tissue image (1024 × 1024 pixels). Error bars are standard deviation (SD). F, Number of nuclear dense areas in 54 images for each class

Our classification analysis of hematoxylin‐stained images indicated that nuclear morphologies are distinct in noncancer and gastric cancers (grades 1‐3), as shown by CA values (Figure 2C). To assess nuclear morphology, we measured two characteristics of the nucleus; area and total intensity (Figure 1A, *right*). Using an image analysis software (Cellomics CellInsight), each measurement region was detected with a fixed size of 1024 × 1024 pixels from original tissue images (Figure 3A). By counting >12 000 nuclei, we found that nuclear area was significantly larger in cancer tissues, compared to noncancerous tissues (Figure 3B), and that signal intensity was also higher in cancer cells (Figure 3C). Because nuclei were densely distributed and sometimes overlapping in cancer tissues, probably due to high growth activities, we then attempted to measure this feature, using the nuclear area that was continuously stained with hematoxylin. We set the software to recognize the hematoxylin‐positive area which was larger than the defined threshold (13 200 pixels), as shown in Figure 3D. The area with clustered nuclei was present prominently in Grades 1 and 2 of gastric cancers, but scarcely in Grade 3 (Figure 3E,F). Summary statistics for the area and total intensity are shown in Table S3, indicating that nuclear morphology is an advantageous parameter for cancer classification.

### 3.4 Expression levels of nuclear ATF7IP/MCAF1 are correlated with H&E images

It has been reported that various nuclear factors^37^, ^38^ and membrane/soluble factors^39^, ^40^ are involved in morphology of cells and tissues. We next investigated biological links between molecular expression and morphological features in gastric cancer tissues, using molecular marker‐based analysis or fact‐driven analysis.

**Figure 4:**
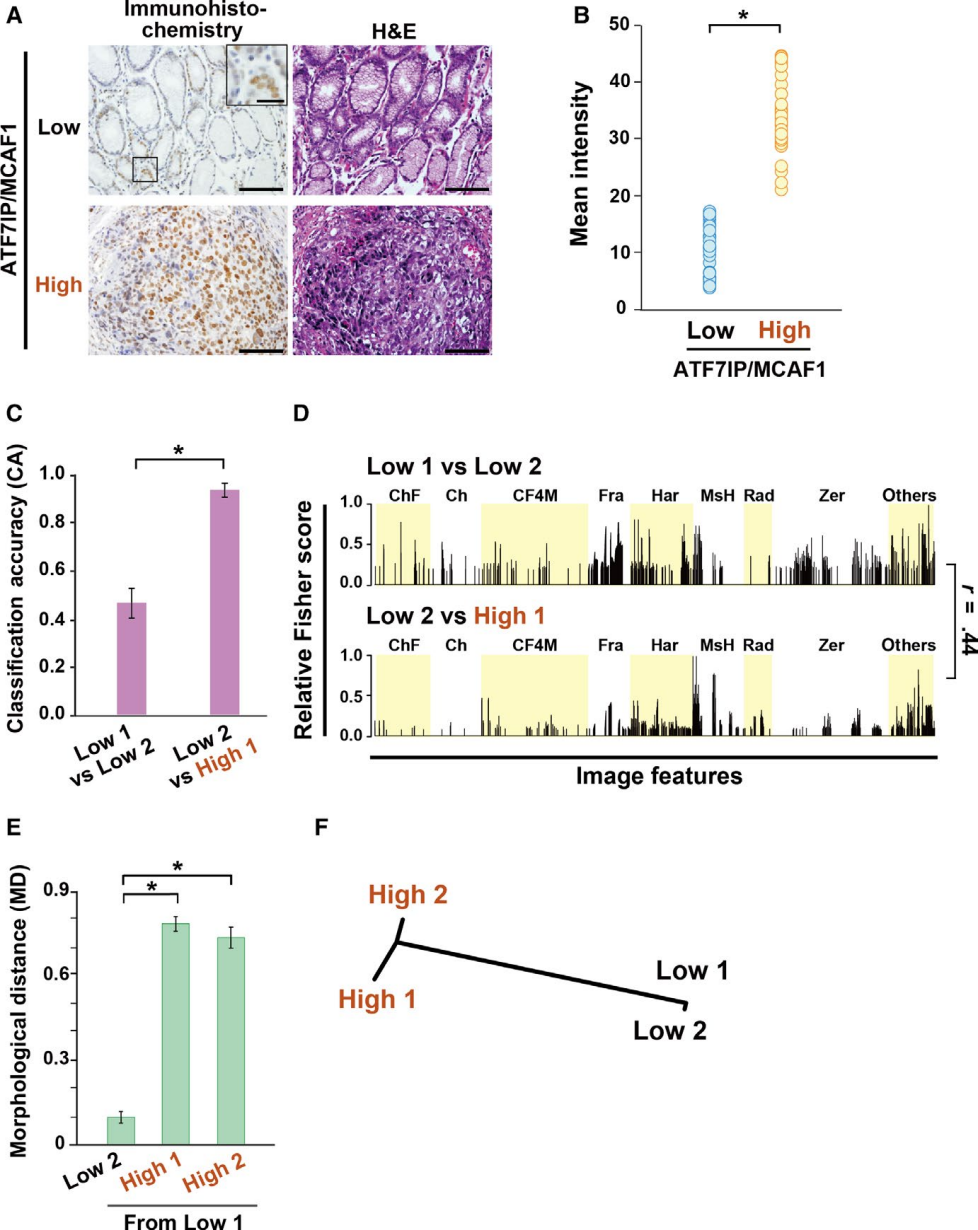
Expression of ATF7IP/MCAF1 is correlated with tissue morphology. A, Representative immunohistochemistry (IHC) images with anti‐ATF7IP/MCAF1 antibodies (*left*) and H&E (*right*) in serial tissue sections. Gastric cancer and adjacent noncancer regions in tissues showed high and low expression of ATF7IP/MCAF1, respectively. Scale bar, 100 µm. Inset shows a few ATF7IP/MCAF1 positive cells in Low region(scale bar, 25 µm). B, Mean intensity of ATF7IP/MCAF1 signals in the IHC image was quantified by ImageJ (n = 32 images in each class). *P* value was calculated using Welch's *t* test (*P < .001) (C) Comparison of CA in indicated binary classifications of H&E stained images. Welch's *t* test (*P < .001). D, Relative Fisher discriminant scores assigned to the 4059 features in binary classification (n = 16 images in each subclass). Specific sets of features were useful to discriminate H&E images between low and high expression of ATF7IP/MCAF1 (weak correlation; r = 0.44). E, MDs from Low 1 of the indicated subclasses. The values represent the average and s.d. of 20 independent cross‐validation tests. Student's *t* test or Welch's *t* test (*P < .001). F, The dendrogram shows the morphological dissimilarities of the H&E images with different expression levels of ATF7IP/MCAF1

**Figure 5:**
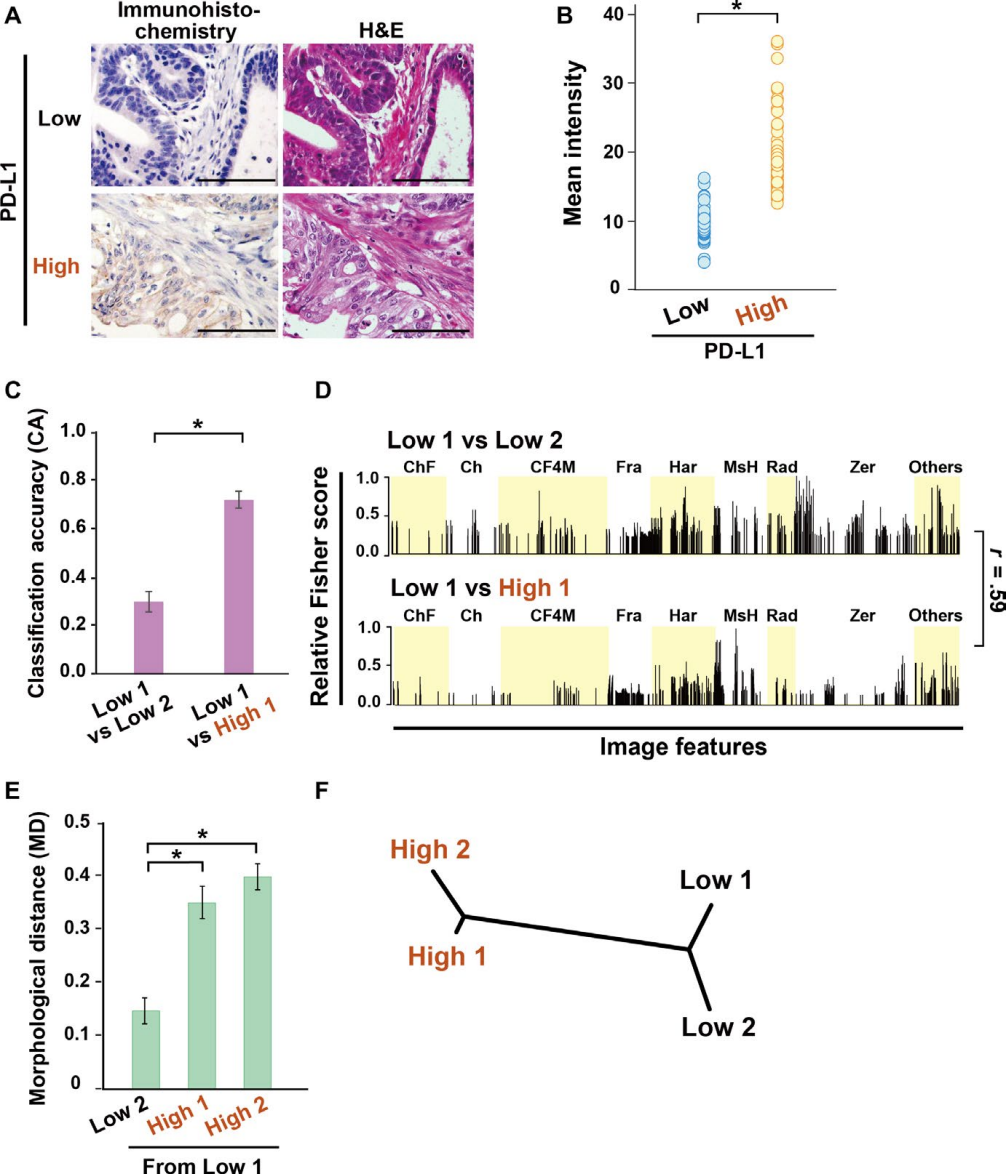
Expression of PD‐L1 is correlated with cancer tissue morphology. A, Immunohistochemistry (IHC) of PD‐L1 and H&E staining for morphological classification. Scale bar, 100 µm. B, Measurement of mean intensity of PD‐L1 signals in IHC image (n = 60 images in each class). *P* values were calculated using Welch's *t* test (*P < .001). C, Comparison of CA in indicated binary classifications of H&E stained images (n = 30 images in each class). Student's *t* test (*P < .001). D, Relative Fisher discriminant scores assigned to the 4059 features for (C) (weak correlation; r = 0.59). E, MDs of the indicated subclasses from the PD‐L1 Low 1. The values represent the mean and SD. *P* values were measured using Student's *t* test (*P < .001). F, The dendrogram shows the morphological dissimilarities of the H&E images with different expression levels of PD‐L1

To examine how H&E images can be classified based on molecular expression, we chose two cancer‐associated proteins: nuclear ATF7IP/MCAF1 and membranous PD‐L1 (Figures 4 and 5). ATF7IP/MCAF1 is an epigenetic factor involved in heterochromatin formation and gene regulation, which is frequently overexpressed in various kinds of tumors including gastric cancers. ATF7IP/MCAF1 functions for either DNA methylation‐based gene repression or the transcription factor Sp1‐mediated gene activation.^36^ On the other hand, PD‐L1 is generally produced by cancer cells to escape immune surveillance, and is a molecular target for cancer immune therapy.^41^, ^42^, ^43^ Previous report showed that the *PD‐L1*gene promoter is regulated by DNA methylation or Sp1 binding in cancer cells.^44^, ^45^ There is the possibility that ATF7IP/MCAF1 may control *PD‐L1* expression via Sp1,as indicated by published ChIP‐seq data of colon cancer (Figure S3A).

We performed both H&E staining and IHC using serial sections of tissue (Table S4). After the section slices were made from a paraffin block and stained, we carefully aligned the H&E with IHC images manually (Figure S3B‐D). We selected 32 sites from H&E images (each 1360 × 1024 pixels) and the corresponding IHC images for ATF7IP/MCAF1 expression. Gastric cancer tissue and adjacent noncancerous regions showed high and low expression of ATF7IP/MCAF1, respectively (Figure 4A, Figure S3E,F). The levels of IHC signals were confirmed by quantification of their signals (Figure 4B). Based on the expression levels of ATF7IP/MCAF1, we then classified H&E images using *wndchrm* to low and high expression of this protein (CA 0.95‐1.00, which shows high accuracy, regardless of image numbers) (Figure S3G), suggesting that gastric cancer tissues as tested can be clearly divided to these two classes. In addition, sensitivity and specificity of ATF7IP/MCAF1 signals were 100% and 98%, respectively (Table S2,*second from lower*). To evaluate the CA between low and high classes of ATF7IP/MCAF1, we arranged subclasses in H&E images (Low 1, Low 2, High 1 and High 2). Low 2 had similarity with Low 1, but significant difference with High 1 (Figure 4C). In addition, alignment of relative Fisher scores indicated weak correlation between the two comparisons (Figure 4D, r = 0.44), suggesting the presence of feature differences. Moreover, each MD from Low 1 in the feature space and the dendrogram showed morphological dissimilarity between low and high classes of ATF7IP/MCAF1 (Figure 4E,F).These suggested that expression levels of this protein are correlated with tissue morphology.

### 3.5 Expression levels of cytoplasmic PD‐L1 are correlated with H&E images

We further investigated whether expression of the membranous protein PD‐L1 in cancer is linked to tissue morphology. We again performed H&E staining and IHC with anti‐PD‐L1 antibodies, using serial sections of tissue microarrays in gastric cancer samples (Figure 5A and Table S5). We quantified IHC signal levels of PD‐L1 staining, grouped into low and high expression of this protein, and further created data‐sets of the corresponding H&E image (1360 × 1024 pixels) (Figure 5B, Figure S4A,B). Furthermore, we evaluated the tumor proportion score (TPS) by counting positively stained cells in 100 cells per image and found that PD‐L1 High showed significantly higher TPS, while PD‐L1 Low had very low TPS(Figure S4C).The H&E images were classified as Low and High PD‐L1, at CA 0.86 using 60 images (Figure S4D). Sensitivity and specificity of PD‐L1 signals were 88% and 84%, respectively (Table S2, *lower*). We confirmed the morphological dissimilarity between PD‐L1 Low and High subclasses as shown by the CA (Figure 5C).The relative Fisher discriminant scores of image features suggested the presence of features responsible for the dissimilarity (Figure 5D,r = 0.59). Each MD from Low 1 in the feature space and the dendrogram showed morphological dissimilarities between Low and High classes of PD‐L1 (Figure 5E,F).

Collectively, these results indicated that the expression of ATF7IP/MCAF1 and PD‐L1 is correlated with tissue characteristics, suggesting that the spatial appearance of the cancer‐associated proteins reflects morphological information of the pathological tissues.

## 4 DISCUSSION

In this study, we found that H&E specimens include potential biological information that distinguishes noncancer and gastric cancer tissues using the target‐free algorithm *wndchrm*. Our fact‐driven image analysis indicated that expression levels of ATF7IP/MCAF1 and PD‐L1 as determined by IHC correspond to tissue morphology in H&E stained images. Thus, quantitative assessments of tissue morphology may reflect molecular changes in cancers, while molecular analyses contribute to morphological evaluation of cancer tissues.

Previous reports indicated that the computational analysis of H&E images assists pathological diagnosis.^12^, ^13^ We also showed that *wndchrm* recapitulated pathological decisions, since the algorithm achieved acceptable classification performance using H&E images of gastric cancers with distinct histological grades (Figure 1).

Both hematoxylin (nuclear) and eosin (cytoplasmic) images contributed to morphological discrimination, indicating that a large quantity of digital information may exist in H&E images (Figure 2). Quantification of nuclear components represented the following characteristics: cell nuclei were smaller, with lower chromatin in noncancerous tissues, while cancer cells exhibited greater heterogeneity, probably due to genetic or epigenetic alterations, together with high nuclear density related to differentiation states (Figure 3). By microscopic diagnosis, it has been shown that nuclear abnormality is a fundamental hallmark of tumor cells and an indicator of patient outcomes in many cancer types.^46^, ^47^ Observations of tissue structural atypia are essential for pathological diagnosis. However, our findings supported that nuclear shape may also be related to the differentiation grades of cancers. Likewise, it has been reported that the cytoplasmic components of cancer and stromal cells have significant features recognized by computational analyses.^48^, ^49^ Using eosin channel data digitally extracted from H&E images, which represent cytoplasmic components, we also obtained good classification accuracy using *wndchrm* (Figure 2).

Although there are some reports that calculated morphological patterns can be ascribed to molecular features,^14^, ^50^ our fact‐driven analysis is a unique strategy for understanding a new framework of image examination. Because image data‐sets are tested by distributions of protein expression, the resulting data indicated that molecular information is linked to morphological features at the tissue level. To validate that molecular differences reflect the cell and tissue morphologies, this study showed that ATF7IP/MCAF1 and PD‐L1 are involved in the molecular background of morphological changes in the tissues studied (Figure 4 and 5).We previously reported that nuclear ATF7IP/MCAF1 functions for heterochromatin formation and gene repression by cooperating with the methylated DNA‐binding protein MBD1 and the histone methyltransferase SETDB1.^51^, ^52^, ^53^ Furthermore, ATF7IP/MCAF1 are overexpressed to maintain telomerase gene expression, together with the transcription factor Sp1 in human cancer tissues such as the stomach, breast, and lung.^36^ Therefore, we assumed that ATF7IP/MCAF1 expression is correlated with changes involving chromatin in nuclei. Gastric cancer tissue and adjacent noncancer regions showed high and low expression of ATF7IP/MCAF1, respectively, suggesting a correlation between the expression levels of this protein and H&E morphology (Figure 4).

Cytoskeletal networks construct cytoplasmic structures and support signal transduction from the extracellular environment to gene expression and chromatin formation in the nucleus.^2^ In fact, soluble factors such as cytokines and growth factors affect cellular structure and function.^39^, ^40^, ^54^ Since monoclonal antibodies against PD‐L1 have been approved for cancer immunotherapy, it is important to investigate effective methods to predict identification of responders using IHC slides.^55^, ^56^ Interestingly, the levels of PD‐L1 expression served to classify tissue morphology in H&E images (Figure 5). These results may indicate that *wndchrm* quantifies unique morphological changes that may be induced by PD‐L1 expression. Considering the contribution of cancer grades to the PD‐L1 classification, we checked the proportion of the grades in PD‐L1 High and Low groups.PD‐L1 High relatively had higher grades, while PD‐L1 Low tended to have lower grades (Table S6), suggesting that histological grades may influence on the classification data in our analysis.

Collectively, our study emphasizes that target‐free image classification and measurements will serve a new work flow to support understanding of molecular mechanisms underlying morphological changes in cells and tissues.

## CONFLICT OF INTEREST

The authors declare no competing interests.

## Data Availability

I confirm that my article contains a Data Availability Statement even if no data is available.
