# Supplementary material for: Computational analysis of morphological and molecular features in gastric cancer tissues
Source: Cancer Med. 2020 Feb 3;9(6):2223–34. doi: 10.1002/cam4.2885 (PMC7064096; doi:10.1002/cam4.2885)
Supplement: Supplementary file 1 [file CAM4-9-2223-s001.docx]

**Supporting Information**

**
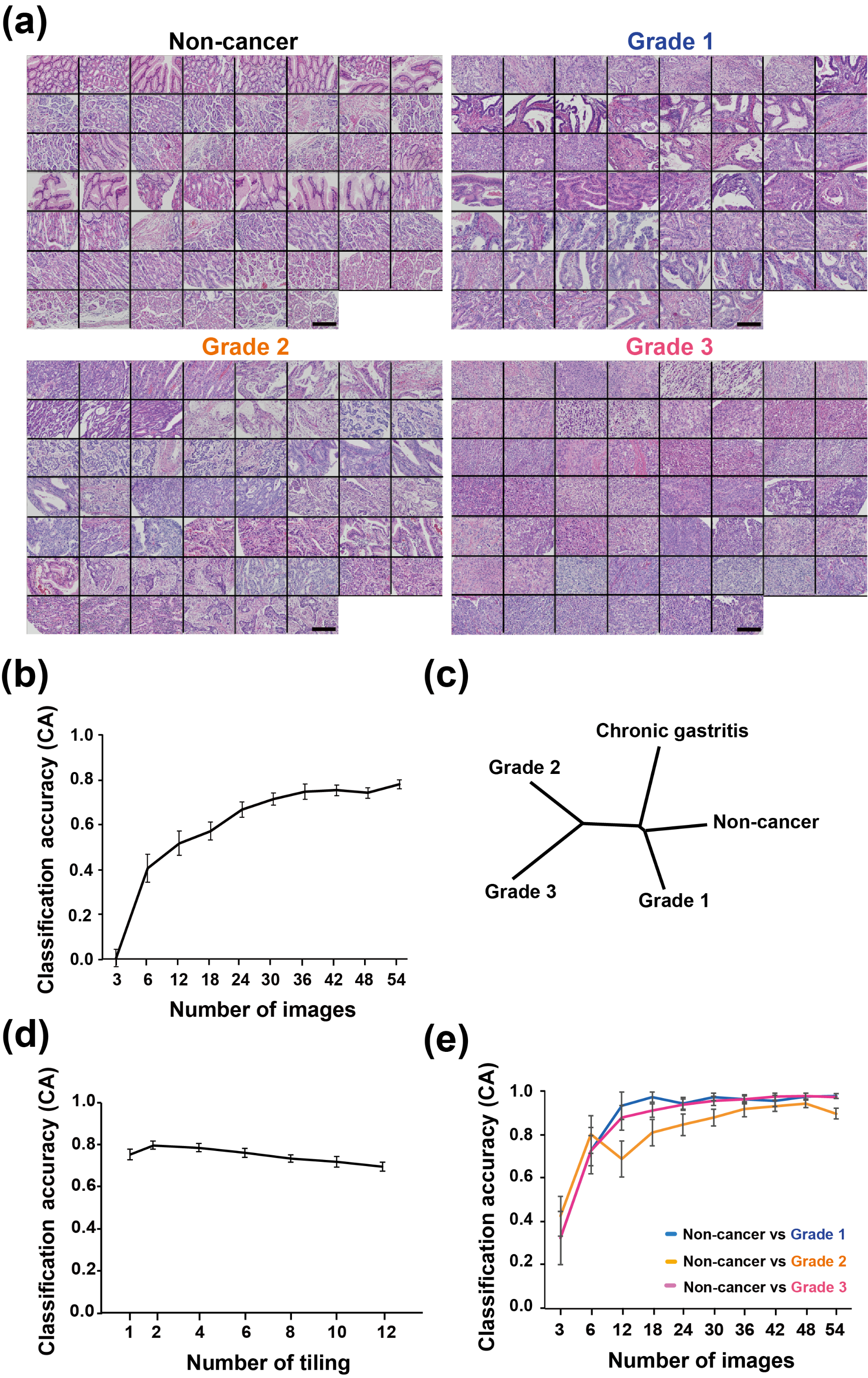
**

**Fig. S1. Use of tissue images for *wndchrm* analysis.** (**a**) Gallery of H&E-stained tissue images for *wndchrm* analysis. Scale bar, 200 μm. (**b**) Classification performances with different numbers of image sets for Non-cancer and Grades 1-3. Classification accuracy (CA) reached plateau with 54 images. Values are the means and s.d. from 20 cross-validation tests. (**c**) A dendrogram shows morphological similarities between 5 classes (n = 20 images in each class), which include non-cancer and chronic gastritis (each two cases) and grades 1 to 3 (each three cases). (**d**) CA analysis using digital tiling of each image. Fifty-four images were used. (**e**) CA of binary classifications between non-cancerous and cancer tissues with indicated grades.


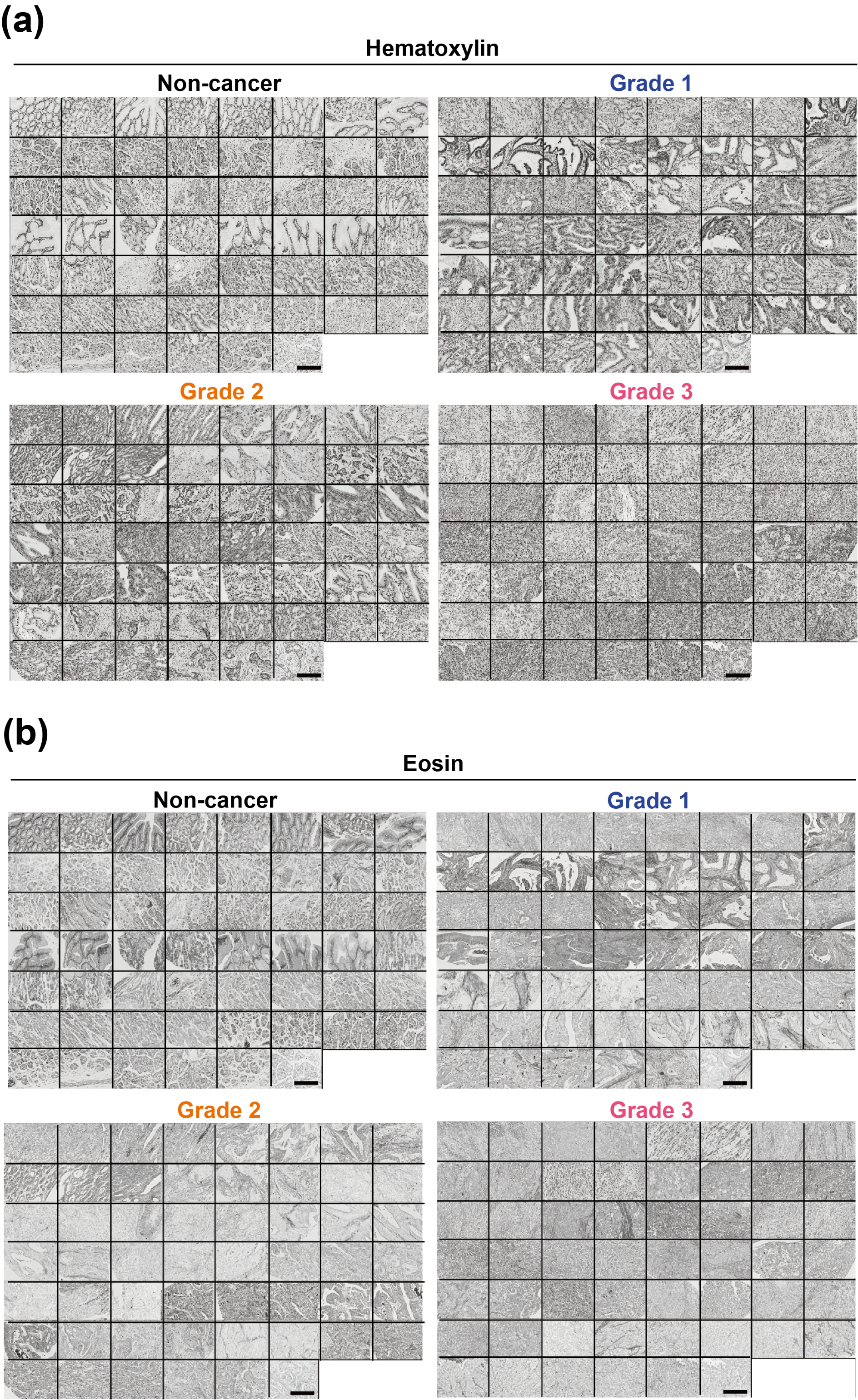


**Fig. S2. Gallery of hematoxylin- or eosin-stained images derived from the H&E images in Fig. S1a.** (**a** and **b**) H&E images were digitally deconvolved to hematoxylin (**a**) and eosin (**b**) channels in gray scales. Scale bar, 200 μm.


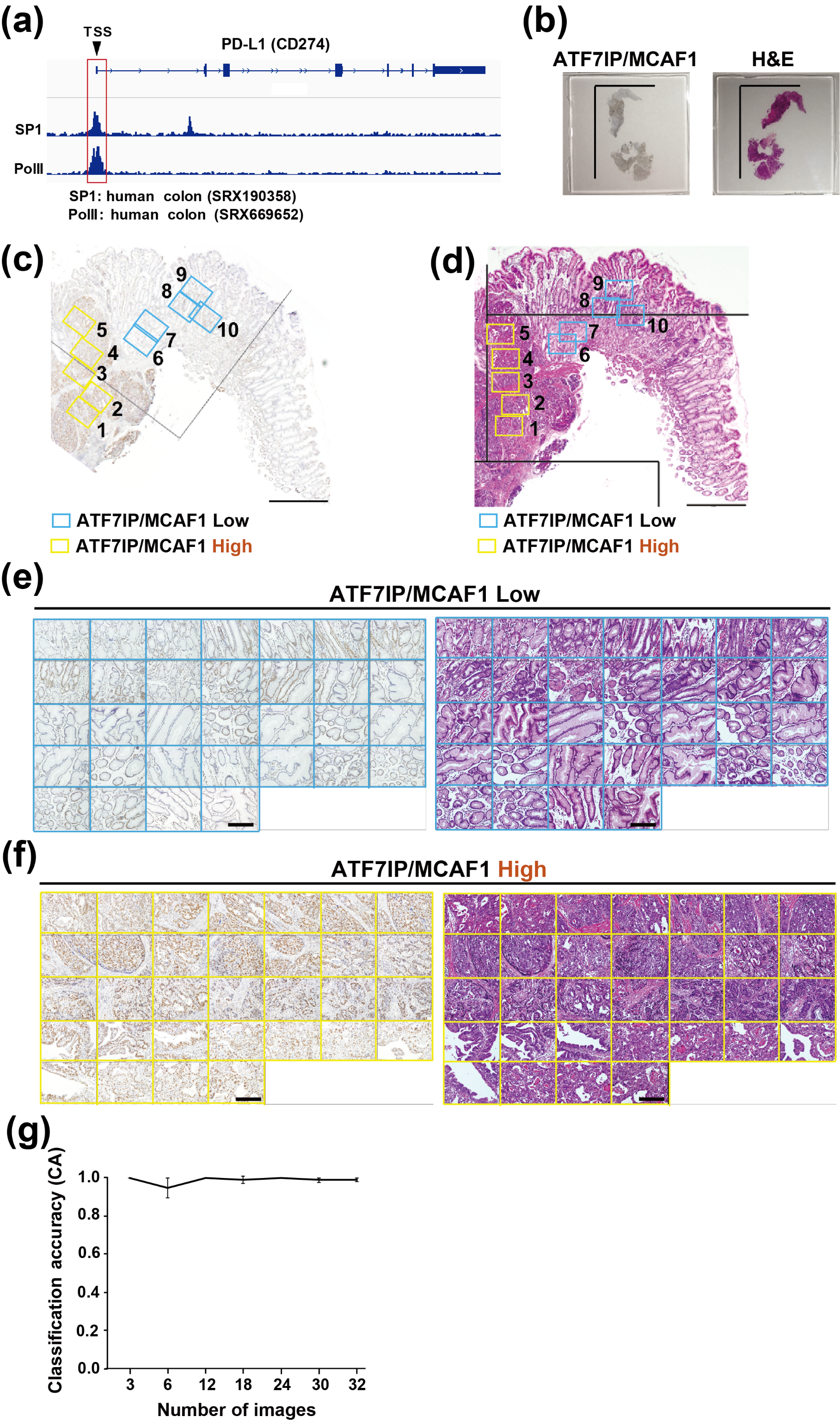


**Fig. S3. Preparation of H&E-stained image data sets corresponding to the ATF7IP/MCAF1 IHC.** (**a**) ChIP-seq signal tracks for Sp1 and Pol II binding sites on *PD-L1* gene promoter region, using ChIP Atlas (https://chip-atlas.org/). (**b**) The zoomed-out images of slides with IHC (*left*) and H&E (*right*). The samples in serial sections were in a rotated position. (**c** and **d**) Matching regions in IHC of ATF7IP/MCAF1 and H&E staining. Each identical region is indicated by the same number. Regions with 1360 × 1024 pixels were manually selected and assigned as ATF7IP/MCAF1 Low (blue) and ATF7IP/MCAF1 High (yellow), according to the ATF7IP/MCAF1 signal intensities in IHC. Scale bar, 1 mm. (**e** and **f**) Gallery of the IHC images (*left*) and their corresponding H&E images (*right*) used for the classifications in **Fig. 4b-f.** Scale bar, 200 μm. (**g**) Classification between ATF7IP/MCAF1 Low and ATF7IP/MCAF1 High classes. CA was constantly high, regardless of image number.


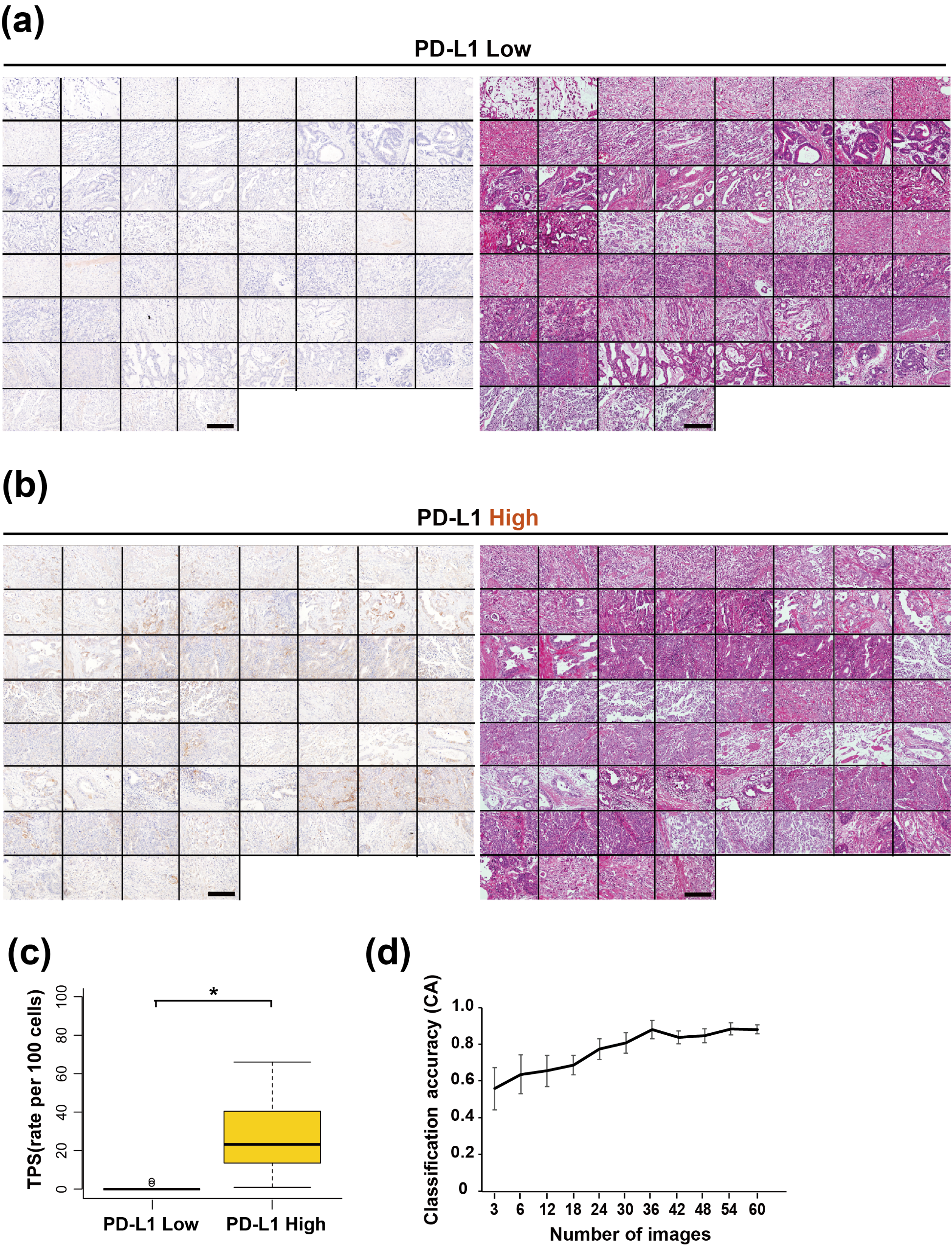


**Fig. S4. H&E-stained image data sets corresponding to the PD-L1 expression levels.** (**a** and **b**) Gallery of images of IHC (*left*) and H&E (*right*), analyzed in **Fig. 5b-f**, using serial sections. The classes of PD-L1 Low (**a**) and PD-L1 High (**b**) had each 60 images. Scale bar, 200 μm. (**c**) Comparison of tumor proportion score (TPS) in low and high classes of PD-L1. P values were calculated using Welch’s t-test (*p < 0.001). (**d**) Classification between PD-L1 Low and PD-L1 High classes. Optimal CA was obtained with 60 images.

**Table S1:** Information on TMA used in Figures 1-3.

**Non-cancer**

| Individual | Age | Sex | Number of images | TMA source |
| --- | --- | --- | --- | --- |
| 1 | 35 | M | 18 | *1 |
| 2 | 83 | M | 19 | *1 |
| 3 | 54 | M | 7 | *2 |
| 4 | - | - | 10 | *3 |

**Grade 1**

| Individual | Age | Sex | Number of images | TMA source |
| --- | --- | --- | --- | --- |
| 1 | 54 | F | 7 | *1 |
| 2 | 36 | M | 5 | *1 |
| 3 | 45 | F | 6 | *1 |
| 4 | 52 | F | 8 | *1 |
| 5 | 62 | M | 6 | *1 |
| 6 | 56 | F | 8 | *1 |
| 7 | 81 | M | 7 | *1 |
| 8 | 35 | M | 7 | *1 |

**Grade 2**

| Individual | Age | Sex | Number of images | TMA source |
| --- | --- | --- | --- | --- |
| 1 | 50 | M | 7 | *1 |
| 2 | 73 | F | 7 | *1 |
| 3 | 72 | M | 7 | *1 |
| 4 | 45 | M | 7 | *1 |
| 5 | 24 | F | 4 | *1 |
| 6 | 61 | M | 5 | *1 |
| 7 | 53 | M | 5 | *1 |
| 8 | 50 | M | 6 | *1 |
| 9 | 80 | M | 6 | *1 |

**Grade 3**

| Individual | Age | Sex | Number of images | TMA source |
| --- | --- | --- | --- | --- |
| 1 | 42 | M | 4 | *1 |
| 2 | 53 | F | 4 | *1 |
| 3 | 55 | M | 4 | *1 |
| 4 | 65 | M | 4 | *1 |
| 5 | 62 | M | 4 | *1 |
| 6 | 75 | M | 4 | *1 |
| 7 | 66 | F | 3 | *1 |
| 8 | 56 | M | 3 | *1 |
| 9 | 54 | M | 3 | *1 |
| 10 | 64 | F | 3 | *1 |
| 11 | 59 | M | 3 | *1 |
| 12 | 35 | F | 3 | *1 |
| 13 | 71 | M | 3 | *1 |
| 14 | 45 | M | 3 | *1 |
| 15 | 74 | M | 3 | *1 |
| 16 | 78 | M | 3 | *1 |

*1: Stomach Tumor Tissue Microarray; BioChain Institute, Inc.; cat. no. Z7020045

*2: AccuMax Array, A700 Ⅱ - Test slide, Various non-cancerous tissue; ISU ABXIS co., LTD; lot. no. #112110611141

*3: MaxArrryTM Human Non-cancer Tissue Microarray Slide; ZYMED Laboratories, Invitrogen Corp; cat. no. 75-4013

**Table S2:** List of sensitivity and specificity related to Figure 1,2,4 and 5.

Classification of non-cancer and grades1-3 cancer using H&E images with 54 images in Figure 1.

| Sensitivity | 92% CI (90 - 94) |
| --- | --- |
| Specificity | 100% CI (98 - 100) |

Classification of non-cancer and grades 1-3 cancer using two types of images with 54 images in Figure 2.

|  | Hematoxylin | Eosin |
| --- | --- | --- |
| Sensitivity | 82% CI (80 - 85) | 89% CI (87 - 91) |
| Specificity | 98% CI (97 - 99) | 98% CI (96 - 99) |

Classification of H&E images corresponding to ATF7IP/MCAF1_low and _high classes with 32 images in

Figure 4.

| Sensitivity | 100% CI (98 - 100) |
| --- | --- |
| Specificity | 98% CI (96 - 99) |

Classification of H&E images corresponding to PD-L1_negative and _positive classes with 60 images in

Figure 5.

| Sensitivity | 88% CI (84 - 91) |
| --- | --- |
| Specificity | 84% CI (80 - 87) |

*CI = 95% confidence interval (binomial test)

**Table S3:** Summary statistics for parameters of the nuclear morphology shown in Figure 3.

**Nuclear area**

|  | Non-cancer | Grade 1 | Grade 2 | Grade 3 |
| --- | --- | --- | --- | --- |
| Number of counts | 12,193 | 14,811 | 15,157 | 16,379 |
| Average | 414 | 502 | 487 | 442 |
| Median | 361 | 428 | 422 | 390 |
| SD | 172 | 235 | 218 | 180 |

**Nuclear total intensity**

|  | Non-cancer | Grade 1 | Grade 2 | Grade 3 |
| --- | --- | --- | --- | --- |
| Number of counts | 12,193 | 14,811 | 15,157 | 16,379 |
| Average | 599,628 | 777,590 | 718,767 | 669,233 |
| Median | 523,584 | 651,888 | 600,496 | 583,824 |
| SD | 291,612 | 418,639 | 397,035 | 329,119 |

**Table S4:** Information on tissue block used in Figure 4.

| Individual | Age | Sex | Cancer cells % | Grade | number of images | TMA source |
| --- | --- | --- | --- | --- | --- | --- |
| 1 | 62 | M | 80 | 3 | 64 | *1 |

*1: Stomach Cancer; formalin-fixed paraffin-embedded tissue, ILSbio Llc, lot.ILS34202PD2

**Table S5:** Information on TMA used in Figure 5.

| Individual | Age | Sex | Number of images | TMA source |
| --- | --- | --- | --- | --- |
| 1 | 52 | F | 5 | *1 |
| 2 | 69 | F | 5 | *1 |
| 3 | 71 | M | 3 | *1 |
| 4 | 68 | F | 5 | *1 |
| 5 | 65 | F | 5 | *1 |
| 6 | 78 | M | 5 | *1 |
| 7 | 67 | F | 4 | *1 |
| 8 | 80 | F | 4 | *1 |
| 9 | 59 | M | 3 | *1 |
| 10 | 83 | M | 3 | *1 |
| 11 | 80 | M | 3 | *1 |
| 12 | 72 | M | 3 | *1 |
| 13 | 68 | M | 3 | *1 |
| 14 | 77 | M | 3 | *1 |
| 15 | 57 | M | 3 | *1 |
| 16 | 68 | M | 3 | *1 |
| 17 | 67 | M | 2 | *1 |
| 18 | 42 | F | 5 | *1 |
| 19 | 61 | M | 2 | *1 |
| 20 | 54 | F | 4 | *1 |
| 21 | 65 | M | 5 | *1 |
| 22 | 62 | M | 4 | *1 |
| 23 | 79 | F | 4 | *1 |
| 24 | 71 | F | 4 | *1 |
| 25 | 57 | M | 4 | *1 |
| 26 | 68 | M | 4 | *1 |
| 27 | 53 | M | 4 | *1 |
| 28 | 68 | F | 4 | *1 |
| 29 | 72 | M | 4 | *1 |
| 30 | 50 | M | 4 | *1 |
| 31 | 78 | M | 2 | *1 |
| 32 | 67 | M | 4 | *1 |

*1: Stomach carcinoma tissue microarray; formalin-fixed paraffin-embedded tissue, US Biomax, Inc., HStm-Ade180Sur-02

**Table S6:** Information on histological grades used in Figure 5.

|  | Ⅰ - Ⅲ | Ⅲ - Ⅳ |
| --- | --- | --- |
| PD-L1 High | 22 | 38 |
| PD-L1 Low | 32 | 28 |

Grade Ⅰ - Ⅲ (lower grades) and Ⅲ - Ⅳ (higher grades)
